# Q fever: Evidence of a massive yet undetected cross‐border outbreak, with ongoing risk of extra mortality, in a Dutch–German border region

**DOI:** 10.1111/tbed.13505

**Published:** 2020-02-20

**Authors:** Volker H. Hackert, Christian J. P. A. Hoebe, Nicole Dukers‐Muijrers, Thomas Krafft, Boris Kauhl, Klaus Henning, Wolfram Karges, Lisa Sprague, Heinrich Neubauer, Sascha Al Dahouk

**Affiliations:** ^1^ Department of Sexual Health, Infectious Diseases, and Environmental Health South Limburg Public Health Service Heerlen The Netherlands; ^2^ Department of Social Medicine and Medical Microbiology Care and Public Health Research Institute (CAPHRI) Faculty of Health, Medicine and Life Sciences Maastricht University/MUMC+ Maastricht The Netherlands; ^3^ Faculty of Health, Medicine and Life Sciences Maastricht University Maastricht The Netherlands; ^4^ Department III Civil Engineering and Geoinformatics Beuth University of Applied Sciences Berlin Germany; ^5^ Institute of Bacterial Infections and Zoonoses Friedrich‐Loeffler‐Institut (FLI) Federal Research Institute for Animal Health Jena Germany; ^6^ Department of Internal Medicine III RWTH Aachen University Hospital Aachen Germany; ^7^ Department of Biological Safety German Federal Institute for Risk Assessment (BfR) Berlin Germany; ^8^ Present address: Department of Medical Care AOK Nordost–Die Gesundheitskasse Potsdam Germany

**Keywords:** communicable disease control, *Coxiella burnetii* infection, international health regulations, one health, outbreaks, Q fever

## Abstract

**Background:**

Following outbreaks in other parts of the Netherlands, the Dutch border region of South Limburg experienced a large‐scale outbreak of human Q fever related to a single dairy goat farm in 2009, with surprisingly few cases reported from neighbouring German counties. Late chronic Q fever, with recent spikes of newly detected cases, is an ongoing public health concern in the Netherlands. We aimed to assess the scope and scale of any undetected cross‐border transmission to neighbouring German counties, where individuals unknowingly exposed may carry extra risk of overlooked diagnosis.

**Methods:**

(A) Seroprevalence rates in the Dutch area were estimated fitting an exponential gradient to the geographical distribution of notified acute human Q fever cases, using seroprevalence in a sample of farm township inhabitants as baseline. (B) Seroprevalence rates in 122 neighbouring German postcode areas were estimated from a sample of blood donors living in these areas and attending the regional blood donation centre in January/February 2010 (*n* = 3,460). (C) Using multivariate linear regression, including goat and sheep densities, veterinary Q fever notifications and blood donor sampling densities as covariates, we assessed whether seroprevalence rates across the entire border region were associated with distance from the farm.

**Results:**

(A) Seroprevalence in the outbreak farm's township was 16.1%. Overall seroprevalence in the Dutch area was 3.6%. (B) Overall seroprevalence in the German area was 0.9%. Estimated mean seroprevalence rates (per 100,000 population) declined with increasing distance from the outbreak farm (0–19 km = 2,302, 20–39 km = 1,122, 40–59 km = 432 and ≥60 km = 0). Decline was linear in multivariate regression using log‐transformed seroprevalence rates (0–19 km = 2.9 [95% confidence interval (CI) = 2.6 to 3.2], 20 to 39 km = 1.9 [95% CI = 1.0 to 2.8], 40–59 km = 0.6 [95% CI = −0.2 to 1.3] and ≥60 km = 0.0 [95% CI = −0.3 to 0.3]).

**Conclusions:**

Our findings were suggestive of widespread cross‐border transmission, with thousands of undetected infections, arguing for intensified cross‐border collaboration and surveillance and screening of individuals susceptible to chronic Q fever in the affected area.

## 1 INTRODUCTION

Following outbreaks in other parts of the Netherlands, the Dutch border region of South Limburg experienced a massive single‐point source outbreak of Q fever related to a local dairy goat farm, counting 253 notified cases of acute human Q fever and an estimated 9,000 undetected infections across the entire region, in 2009 (Hackert et al., 2012; van Leuken et al., 2013). Q fever is a bacterial zoonosis caused by *Coxiella burnetii*, transmitted to humans from infected ruminants, primarily by the airborne route (Cutler, Bouzid, & Cutler, 2007; Maurin & Raoult, 1999; Parker, Barralet, & Bell, 2006). In acute disease, flulike illness is usually prominent. Most Q fever infections are mild or asymptomatic and self‐limiting (Hackert et al., 2012; van der Hoek et al., 2012). However, a small percentage of infected individuals may develop chronic Q fever, which often goes unnoticed for years after infection but is usually fatal if left untreated. In addition, a substantial proportion of infected individuals may suffer symptoms referred to as Q fever fatigue syndrome, which may persist for years, with major health‐related consequences (Morroy et al., 2016). In South Limburg, the distribution of notified cases of acute human Q fever followed a west–east gradient of decreasing incidence from the source, following westerly winds predominant at the time of the outbreak, towards and up to the Dutch–German border (Hackert et al., 2012). In the same year, only six cases of acute Q fever (notifiable under German law) were reported from the entire German federal state of North Rhine‐Westphalia, none of whom was a resident of the two districts bordering South Limburg (Heinsberg and Aachen). In the five years preceding the outbreak (2004–2008), a total of 42 cases were reported from North Rhine‐Westphalia, just one of whom lived in Aachen (2006). In 2010, the year following the outbreak, North Rhine‐Westphalia counted a total of 14 cases, only 2 of whom were from Aachen (Robert Koch Institute, 2016). Whereas these data suggest that cross‐border transmission from South Limburg to neighbouring German counties was negligible, it is rather unlikely that airborne transmission stopped short of the Dutch–German border. A Belgian study suggests that a limited degree of transmission took place in westerly direction across the Dutch–Belgian border, but does not quantify the extent of transmission (Naesens et al., 2012). Recent spikes of newly detected cases of late chronic Q fever in the Netherlands, with a high burden of extra mortality, show that the Dutch epidemic is far from over and should be seen as an ongoing public health concern with reason for unabated alertness (Radboud University Medical Center, 2018). This may be even more the case in a population unknowingly exposed to Q fever, where the risk of delayed or overlooked diagnosis of chronic Q fever may be even higher. We therefore aimed to assess the scope and scale of any undetected cross‐border transmission to neighbouring German counties associated with the regional outbreak in South Limburg.

## 2 MATERIALS AND METHODS

### 2.1 Study area, study population and study period

The Meuse–Rhine Euroregion provided the administrative background for our study (Wikipedia contributors, 2017). Geographically, it covers an area of about 11,000 km^2^ around the city corridor of Aachen (North Rhine‐Westphalia, Germany), Maastricht (South Limburg, Netherlands), Hasselt (Limburg, Belgium) and Liège (Liège, Belgium).

The Dutch study area was defined by the approximate catchment area of a large regional general hospital (346 km^2^, 12 municipalities and 308,410 inhabitants).

The adjacent German study area was defined by the 122 postcodes of individual residents from North Rhine‐Westphalia who donated blood at the RWTH Aachen University Hospital Blood Donation Centre in the first two months of 2010. For a summary of statistics regarding study area and study population, see Table 1. Of the 3,460 included German blood donors, the majority (*n* = 3,083, 89.1%) lived in postcode areas whose centroid was located within 40 km from the outbreak farm. The study was conducted from February 2009 (when first abortions were registered on the outbreak farm) to February 2010, when (a) no more incident human cases of acute Q fever were reported in the Dutch study area; (b) culling of pregnant dairy goats to prevent further transmission during the upcoming 2010 lambing season had been finalized; and (c) inclusion of German blood donors (January and February 2010) ended.

**Table 1:** Study area characteristics in radial 20‐km distance classes from the index dairy goat farm in South Limburg, Netherlands

	Distance from outbreak farm				
	0–19 km	20–39 km	40–59 km	≥60 km	
**Population**	***n***	***n***	***n***	***n***	**Total**
Dutch area^a^	308,410	0	0	0	308,410
German area^b^	241,131	478,897	302,654	1,957,216	2,979,898
Total (Dutch and German area)	549,541	478,897	302,654	1,957,216	3,288,308
**Surface area**	**km^2^**	**km^2^**	**km^2^**	**km^2^**	**Total**
Dutch area	927	0	0	0	927
German area	768	1,619	2,478	8,270	13,135
Total (Dutch and German area)	1,695	1,619	2,478	8,270	14,062
**Postcode areas**	***n***	***n***	***n***	***n***	**Total**
Dutch area	70	0	0	0	70
German area	8	17	16	81	122
Total (Dutch and German area)	78	17	16	81	192
**Mean postcode size**	**km^2^**	**km^2^**	**km^2^**	**km^2^**	
Dutch area	13.2	0	0	0	
German area	109.7	101.2	154.9	104.7	
**Mean livestock density per km^2^**	***n***	***n***	***n***	***n***	
Goats	5.6	0.7	0.5	4.4	
Sheep	12.6	14.2	8.5	6.0	
Cattle	53.6	99.1	65.8	55.6	

a Eastern South Limburg, defined by catchment area of local general hospital.

b Catchment area of RWTH Aachen University Hospital Blood Donation Centre, including 122 postcodes counting at least one resident visiting the centre in January/February 2010.

### 2.2 Epidemiological investigation

**Figure 1:**
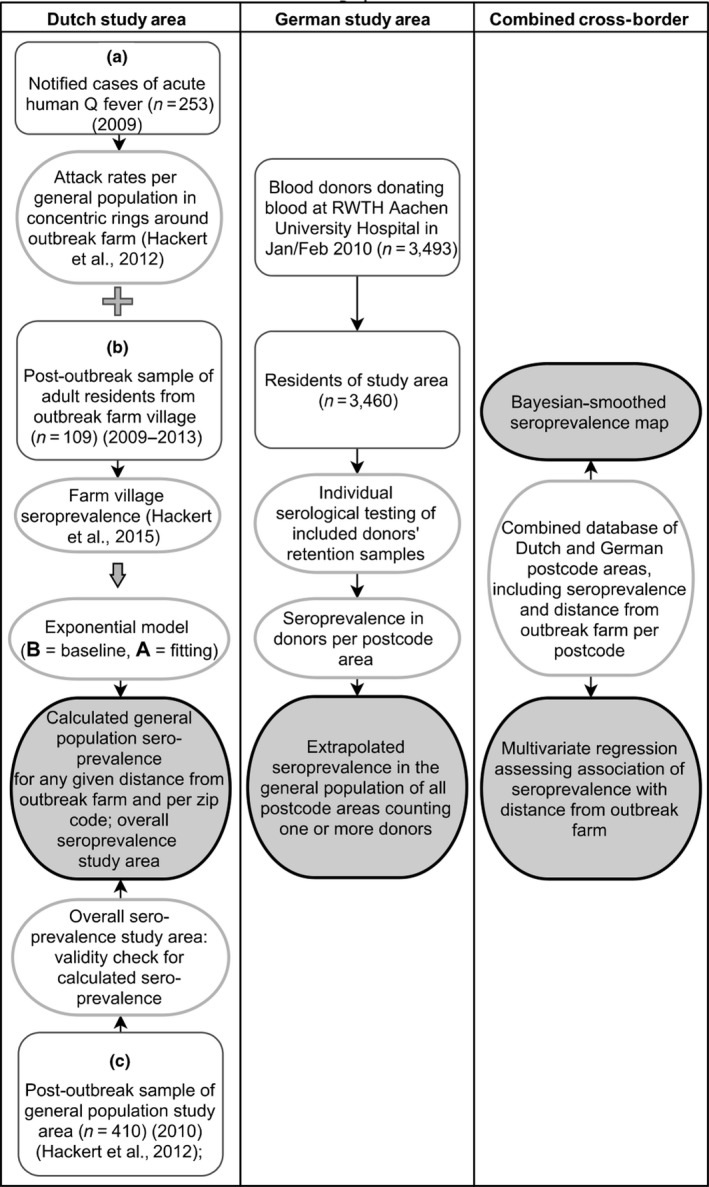
Outline of population samples and study design by study area (Dutch study area, German study area, combined cross‐border study area)

To assess seroprevalence of Q fever in the Dutch, German and combined cross‐border study area in relation to distance from the Dutch outbreak farm, we used various population samples and methods of analysis. An outline is given in Figure 1.

### 2.3 Dutch study area

The outbreak affected a population largely naive to Q fever, according to a serological survey from the year predating the outbreak (2008). Human Q fever cases notified to the PHS South Limburg in 2009/10 (*n* = 253) were scattered downwind from the outbreak farm, following a gradient of declining attack rates in easterly direction from the source up to the Dutch–German border (Hackert et al., 2012). Using SPSS's curve fitting tool, we derived a curve of best exponential fit from aforementioned gradient, corresponding to the following model: 469.074733 * EXP (−0.321415 * [distance to outbreak farm in km]). Q fever seroprevalence in a convenience sample of adult residents from the outbreak farm's township (*n* = 120, aged ≥ 18 years, mean household distance from outbreak farm = 2.7 km) served as a baseline estimate for the calculation of seroprevalence rates across the entire distance from the outbreak farm up to the Dutch–German border according to our exponential model (Hackert et al., 2015). Underlying was the assumption that infections followed the same geographical gradient as notified cases. Seroprevalence in this township sample was age‐ and sex‐matched within 10‐year age strata according to the demographic distribution in our sample of German blood donors. To assess validity of our exponential model, we compared the calculated cumulative seroprevalence for the entire Dutch area with seroprevalence observed in a post‐outbreak convenience sample of adults from the same area (Hackert et al., 2012).

### 2.4 German study area

A total of 3,460 out of 3,493 blood donors (99.1%), who visited the Blood Donation Centre in January/February 2010, were residents of North Rhine‐Westphalia, while *n* = 39 were residents of remote German federal states and consequently excluded from our study. Seroprevalence was estimated using retention sample sera from the 3,460 included blood donors. The detection of IgG or IgM phase II antibodies by ELISA or of *C. burnetii* DNA by qPCR identified positive sera. Minimum age for blood donations in Germany is 18 years. Apart from standard exclusion criteria relating to donor blood safety, additional criteria were applied during the study period to exclude donors with increased risk of recent or acute Q fever infection (contact with livestock, such as cattle, sheep, goats, rabbits and ducks, or their excrements, over the preceding 5 weeks; living in the vicinity of a livestock holding; and signs or symptoms of fever, sweats, nausea, vomiting, diarrhoea, malaise or headaches in the five weeks preceding donation). Cases of acute Q fever reported to the German public health authorities were retrieved from the publicly accessible notification database (SurvStat) of the Robert Koch Institute (Robert Koch Institute, 2016). For reasons of privacy protection, demographic blood donor information was limited to residential postcode and 10‐year age group. Based on postcode centroids as a proxy for residential address, GIS was used to map the geographical distribution of seropositive and seronegative donors, to determine seroprevalence in postcode areas and to extrapolate the seroprevalence to the general population in these areas.

### 2.5 Combined Dutch–German cross‐border study area

**Figure 2:**
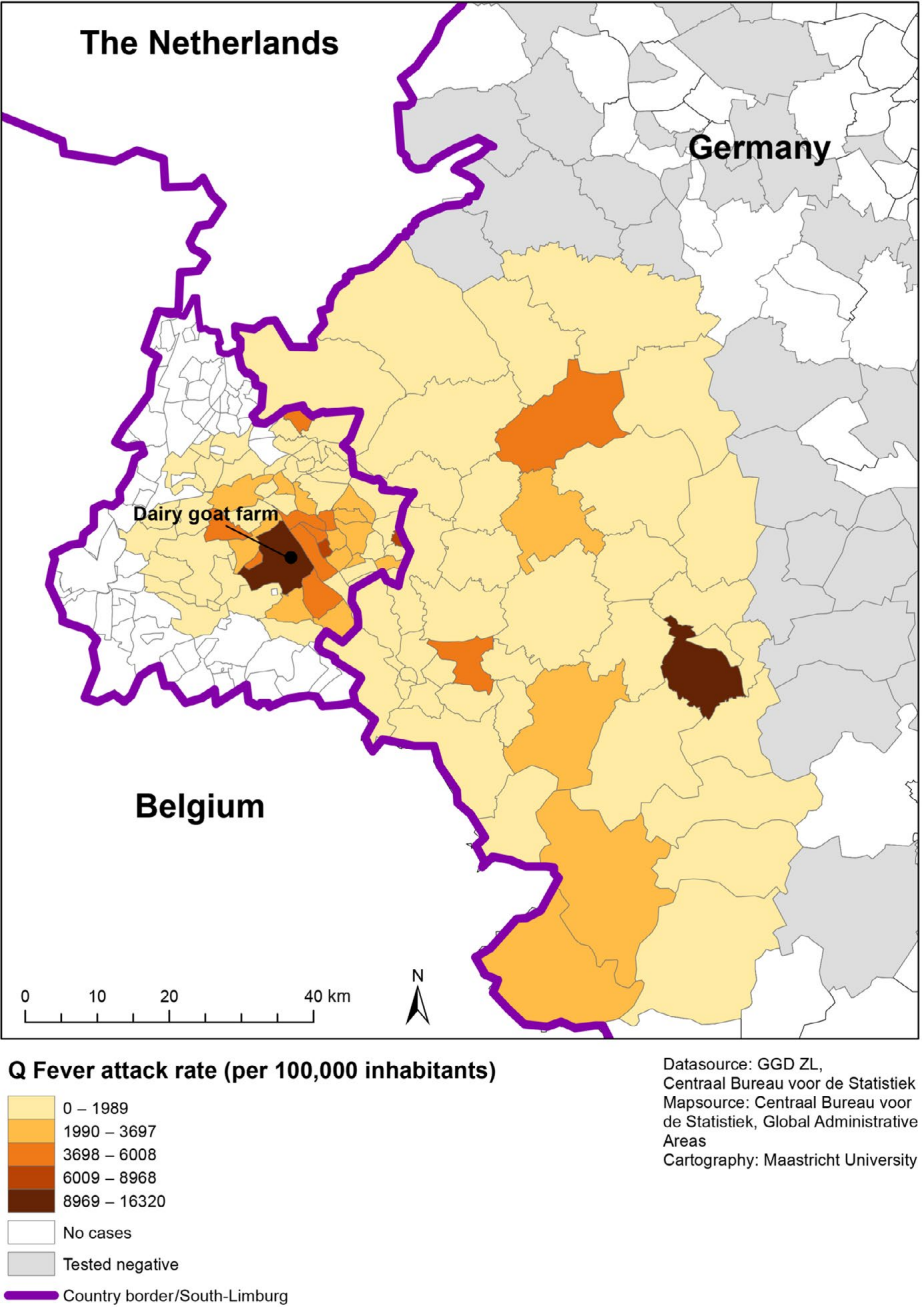
Bayesian‐smoothed extrapolated Q fever seroprevalence rates in the Dutch–German cross‐border region by postcode area (dairy goat farm = location of the outbreak farm in South Limburg, Netherlands)

A regression model, including distance zones of 20 km as dummy variables, adjusting for goat and sheep densities, veterinary Q fever notifications and sampling rates (i.e. number of individuals tested per 100,000 population in each postcode area), was used to assess the geographical relationship between seroprevalence (assumed to represent incidence rate of infection) and exposure dose (approximated by residential distance from the outbreak farm). In addition, we applied our fitted model, derived from the geographical distribution of attack rates in the Dutch study area, to predict seroprevalence rates in the German study area by distance from the outbreak farm, and tested the correlation between predicted and observed rates. We used Spatial Empirical Bayes Smoothing (where estimates per postcode are weighted against estimates in neighbouring areas sharing a common edge or border) to visualize our data by creating a smoothed map of seroprevalence rates in the combined Dutch–German cross‐border region (Figure 2). Computations were carried out in OpenGeoDa 1.2.0 (Anselin, 2005).

We used multivariate linear regression to assess the relationship between postcode seroprevalence rates, log‐transformed for better visualization and radial distance from the outbreak farm in 20‐km zones. Goat and sheep densities, veterinary Q fever outbreaks and sampling densities (per 100,000 population) were included as covariates. A priori, we chose to include goat and sheep densities as covariates in our multivariate regression model, irrespective of univariate linear regression outcome, given their important role as reservoirs and sources of human Q fever. Variables did not show collinearity.

### 2.6 Laboratory investigation

Laboratory procedures performed during the Dutch outbreak were described previously (Hackert et al., 2012). All retention samples of the German blood donors and the Dutch serum samples were screened for anti‐*Coxiella* phase II IgG according to manufacturers' protocols (Serion ELISA classic, Institut Virion/Serion GmbH, Würzburg, Germany). A selection of negative sera (*n* = 128) and all positive or indeterminate sera was additionally tested for anti‐*Coxiella* phase II IgM (Serion ELISA classic, Institut Virion/Serion GmbH, Würzburg, Germany) and for the presence of *Coxiella* DNA using qPCR. We essentially applied the qPCR assay described elsewhere with a slightly modified TaqMan probe (Klee et al., 2006).

### 2.7 Veterinary data

Human Q fever cases from 2009 were linked to a single dairy goat farm in South Limburg, whose nearest distance to the Dutch–German border in a south‐eastern direction was 7.0 km (Hackert et al., 2012; van Leuken et al., 2013).

Municipality‐level data on goat, sheep and cattle population densities (number of animals per km^2^) in the Dutch study area were retrieved from the National Bureau of Statistics (Statistics Netherlands, Statline, 2009). Comparable animal data in the German study area were obtained from the statistical bureau of North Rhine‐Westphalia, according to its 2010 livestock census (Information und Technik, Nordrhein‐Westfalen, Geschäftsbereich Statistik, Statistische Berichte, Viehaltungen und Viehbestände am 1. März 2010, Ergebnisse der Landwirtschaftszählung). These data were available on a district level only. Confirmed (but not suspect) cases of Q fever in ruminants (goat, sheep and cattle) are notifiable under German law. Data on Q fever notifications in ruminants were retrieved from the Animal Disease Reporting System (TSN), the standard electronic system for registration of all notifiable and reportable animal diseases in Germany, for the entire German study area and study period (Probst, 2010).

### 2.8 Statistical analysis

Statistical analyses were performed using IBM SPSS Statistics, version 21 (IBM Inc.). We performed bootstrapping on our estimates using non‐linear regression to obtain more robust confidence interval estimates. We report B values along with their 95% confidence intervals derived from bootstrapping.

## 3 RESULTS

### 3.1 Overall seroprevalence in the Dutch–German cross‐border region

Our smoothed map shows a large area of adjacent postcodes affected by human Q fever surrounding the outbreak farm, spreading over distances of more than 40 km into the German border region.

Baseline seroprevalence in the outbreak farm township, adjusted for age and gender, was 16.1%. Calculated mean seroprevalence for the Dutch study area, derived from the observed gradient of attack rates, was 3.6%, close to the 4.4% observed in the age‐ and gender‐adjusted general population sample from the Dutch study area dating from 2010. The difference between mean calculated and mean observed seroprevalence was statistically not significant by *t* test (*p* = .26).

Observed seroprevalence in blood donors from the German study area was 0.9% (31/3,460) overall, extrapolating to a total of 11,308 infections in those postcode areas with at least one seropositive blood donor. Among the positive blood donors, 61.3% (19/31) had a serological profile (anti‐*Coxiella* phase II IgM) indicative of a fresh or recent infection and/or were *Coxiella* DNA positive: 13 donors had a solitary anti‐Coxiella phase II IgM (one of whom was also qPCR positive) and six were positive for both phase II IgM and IgG, while 15 had a solitary phase II IgG response.

### 3.2 Seroprevalence over 20‐km distance classes from the outbreak farm

**Figure 3:**
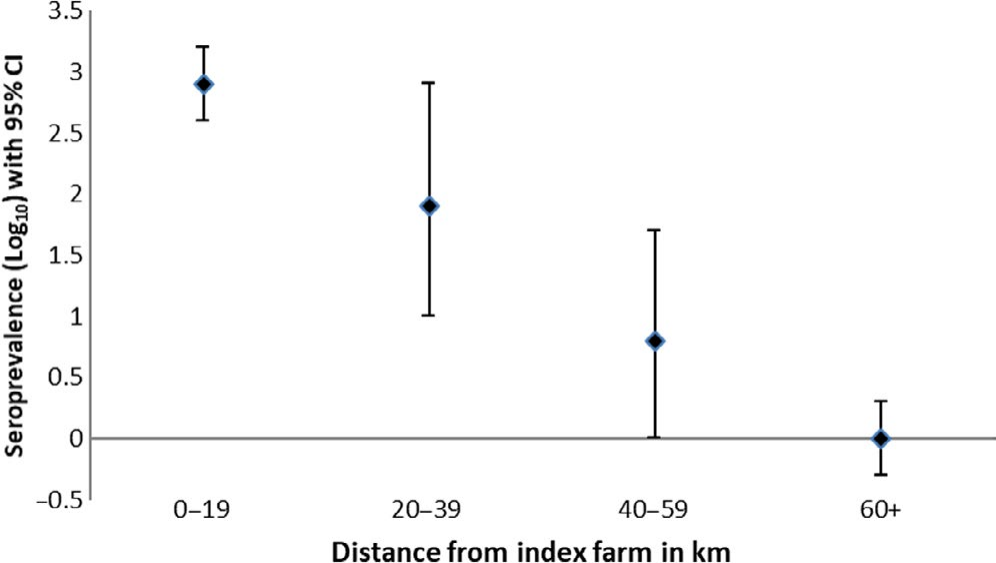
Log‐transformed seroprevalence in radial 20‐km distance classes from outbreak farm, point estimates based on multivariate linear regression including residential distance from outbreak farm, livestock densities (sheep and goats) and screening rates as predictors, with 95% confidence intervals derived from bootstrapping, including the ‘outlier’ postcode in the German study area [Colour figure can be viewed at wileyonlinelibrary.com]

Seroprevalence rates across radial 20‐km distance classes declined with increasing distance from the outbreak farm (Table 2 and Figure 3), comparable with log‐transformed estimates in our multivariate linear regression model. Multivariate analysis was performed twice, with and without inclusion of a high‐seroprevalence postcode on the German side, located at a distance of 54 km from the outbreak farm and figuring as a dark‐coloured ‘hotspot’ in our smoothed map. This postcode counted six donors, one of whom was seropositive (seroprevalence = 16.7%). Given that seroprevalence in surrounding postcodes was low (i.e. 1.6% for the entire district including 18 postcode areas and 257 donors), and there were no reports of Q fever in livestock from the district during the study period, high seroprevalence in this postcode was unlikely to reflect locally acquired infection. This ‘outlier’ had limited impact on our findings.

**Table 2:** Blood donor test results and Q fever seroprevalence rates in postcode area populations in radial 20‐km distance classes from the index dairy goat farm in South Limburg, Netherlands

	Distance from outbreak farm (km)			
	0–19	20–39	40–59	≥60
*Blood donors tested (n)*	1,268	1,815	227	150
*Blood donors positive for Q fever (n)*	12	16	3	0
*Seroprevalence*				
Tested blood donors (per 1,000)	9.5	8.8	1.3	0
Postcode area populations (per 100,000)				
Including ‘outlier’ postcode				
Mean (per postcode area)	2,302^a^	1,122	1,447	0
Log10 crude	2.8	1.7	0.7	0
Log10 adjusted^b^	2.9 (2.6–3.2)	1.9 (1.0–2.9)	0.8 (0.0–1.7)	0.0 (−0.3–0.3)
Excluding ‘outlier’ postcode				
Mean (per postcode area)	2,302^a^	1,122	432	0
Log10 crude	2.8	1.7	0.5	0
Log10 adjusted^b^	2.9 (2.6–3.2)	1.9 (1.0–2.8)	0.6 (−0.2–1.3)	0.0 (−0.3–0.3)

a Based on calculated seroprevalence in the Dutch area, derived from our exponential model and baseline sample from outbreak farm township general population and observed seroprevalence in German blood donors.

b Adjusted for goat and sheep density, veterinary Q fever notifications and sampling density per 100,000 population, with 95% confidence intervals derived from bootstrapping (in brackets).

### 3.3 Predicted versus observed seroprevalence in the German study area

Mean predicted log‐transformed seroprevalence for all postcode areas included in our German study area, based on the gradient of attack rates of acute Q fever observed in the Dutch study area, was 0.27 (untransformed 27 per 100,000), while mean observed log‐transformed seroprevalence was 0.43 (untransformed 262 per 100,000). This difference between predicted and observed log‐transformed seroprevalence estimates was statistically not significant by *t* test (*p* = .08). Correlation between predicted and observed log‐transformed values was 0.48 using Pearson correlation (*p* < .001) and 0.49 using Spearman's rho (*p* < .001).

## 4 DISCUSSION

Transmission of Q fever over distances of at least 30 km has been described before (Eldin et al., 2017; Tissot‐Dupont, Amadei, Nezri, & Raoult, 2004). Our study, however, is first to provide evidence of large scale yet undetected long‐distance transmission of Q fever in a Dutch–German cross‐border context, associated with the 2007–2010 Q fever epidemic in the Netherlands. Presumed transmission into neighbouring German counties took place over distances of 40 km or more from a single dairy goat farm located in South Limburg, the southernmost part of the Netherlands.

Our study suggests that cross‐border transmission, in spite of evidence for massive numbers of infections dispersed over a wide geographical area, went largely undetected, posing susceptible patients at risk of long‐term sequelae, most notably chronic Q fever, due to delayed diagnosis, missed diagnosis or misdiagnosis. Extrapolating from our sample of blood donors, the estimated number of unreported infections in the affected German border area may have been as high as 11,000.

Our data suggest that the risk of Q fever infections going undetected was higher on the German than on the Dutch side of the border, with ascertainment rates (i.e. numbers of notified adult cases vs. estimated numbers of infections) being at least 10 times lower on the German side (Hackert et al., 2015, 2012). Based on unpublished hospital data from South Limburg, with 17 confirmed cases of Q fever, we estimate that the 10‐year incidence of chronic Q fever is 1 in 1,000 infected individuals, with a case fatality rate of approximately 70%. As yet unpublished data from the German cross‐border area, showing high numbers of overlooked or misdiagnosed chronic Q fever infections in hospitalized patients, lend further support to our findings.

The magnitude and relevance of this ongoing public health concern are underlined by recent figures from the Netherlands. As of 2018, the Netherlands had counted a total of 519 chronic Q fever patients since 2007. Of these, 86 patients had died, 21 of whom between 2016 and 2018 alone (Radboud University Medical Center, 2018). While incidence and prevalence of Q fever fatigue syndrome have been registered neither regionally nor nationally, studies suggest that approximately 20% of Q fever patients are affected by long‐term fatigue persisting for up to 20 years, adding to the disease burden related to the Dutch epidemic (Morroy et al., 2016).

Seroprevalence in our study declined exponentially with increasing distance from the outbreak farm across four 20‐km zones. The observed west–east gradient is compatible with dispersal of *C. burnetii* from the farm, given that transmission of Q fever is usually airborne, and westerly winds were predominant in the study area at the time of Q fever‐related abortions in pregnant goats on the outbreak farm.

Observed seroprevalence rates in the German study area were higher than rates predicted by our exponential model, with moderate statistical correlation between the two. Alternative sources, such as contaminated manure, wildlife reservoirs and sheep flocks that migrate over longer distances and have been shown to carry *C. burnetii* in clinically inconspicuous animals, may have contributed to human infections in the German border region, but these phenomena would not explain the geographical distribution observed (Hermans, Jeurissen, Hackert, & Hoebe, 2014; Hilbert et al., 2012; Webster, Lloyd, & Macdonald, 1995). While reports of Q fever in livestock in the German border region during the study period may implicate cattle as potential sources, a recent study found that human contact with sheep and goats, rather than cattle, was a consistent risk factor in human outbreaks (Georgiev et al., 2013; Verso et al., 2016). Goat and sheep densities in the German cross‐border area, however, were rather low (see Table 1). While goats and sheep may have contributed to our seropositive findings, they thus seem unlikely sources for large numbers of human infections spread over a wide area. In addition, our data did not reveal any plausible local source other than the outbreak farm.

Nevertheless, while the observed west–east gradient of seroprevalence rates in the population is consistent with airborne dispersal of *C. burnetii* from the index farm, we cannot exclude other routes of transmission contributing to the observed geographical distribution of infections. For example, active human mobility in the vicinity of dairy goat farms with a history of Q fever‐related abortion waves has been shown to increase the risk for positive Q fever serology (Klous et al., 2019). The province of Limburg is the most popular day‐travel destination for German tourists (ZKA Consultants & Planners, 2012). Moreover, 1.7% of workers in the region are cross‐border commuters from Germany. Thus, an unknown proportion of blood donors may have acquired the infection through travel or transit through South Limburg. Blood donors living closest to the border could be expected to have the highest risk of infection related to airborne transmission and travel alike, as they would be most likely to undertake day trips or commute into neighbouring South Limburg. Human cross‐border movement as a contributing factor would not diminish the relevance of our findings or change the fact that hidden transmission revealed by our data would have important implications for cross‐border communicable disease control in terms of alerting members of the public and the medical profession of potential risks of exposure and associated health hazards.

Under‐ascertainment and under‐reporting of Q fever are usually attributed to its mild and non‐specific clinical presentation. Primary infections are often mild, sometimes resembling a common cold, or asymptomatic, diagnosed only retrospectively through systematic testing (Eldin et al., 2017). This phenomenon is mirrored by a growing number of studies where seroprevalence rates in the population exceed reported cases of symptomatic Q fever. One such study from Denmark showed a rate of 64% of asymptomatic primary infections, while a study from Italy reported 30 seropositive individuals with no related episodes of respiratory or febrile disease (Bacci et al., 2012; Verso et al., 2016). A recent study from Spain found fever—usually considered the hallmark of symptomatic infection—to be absent in almost a third of 39 Q fever positive cases, even though all cases without fever had pneumonia. Interestingly, a systematic review by the same authors reported the absence of traditional risk factors, most notably animal exposure, in a majority of almost 1,500 included Q fever cases, with as much as 60% of cases living in urban areas, raising the possibility of airborne and other routes of transmission in these cases (Alende‐Castro et al., 2018).

Seroprevalence studies from the Netherlands following the Dutch 2007–2010 Q fever epidemic revealed incident Q fever infections to exceed notified infections by factors of ten and higher (Hackert et al., 2015,2012; Hogema et al., 2012; van der Hoek et al., 2012,). A syndromic surveillance study that retrospectively identified three clusters of lower respiratory infections dating from 2005 and 2006 plausibly linked to the Dutch epidemic appears to confirm that even clusters of more severe disease may easily be missed by physicians (van den Wijngaard et al., 2011). In the case of the cross‐border outbreak we describe here, limited attention paid to the outbreak in South Limburg by regional German media, health professionals and members of the public may have influenced peoples' help‐seeking behaviour and resulted in a low index of suspicion towards Q fever in clinical cases, as well as misperceptions about the outbreak's potential for widespread cross‐border transmission. Also, since goat husbandry is uncommon in Germany, there may have been a mistaken belief that the epidemic was just a domestic Dutch problem, reinforced by unfamiliarity with *C. burnetii's* potential for airborne transmission over long distances.

Our study has limitations. While we had data for the Dutch region showing that pre‐outbreak seroprevalence in 2008 was as low as 0.5%, we had no pre‐outbreak data for the German study area. Seropositive findings in the blood donors may thus reflect past exposure to sources other than the Dutch outbreak farm. However, more than 60% of the positive blood donors had serological profiles indicative of acute or recent infection, arguing for a close temporal association with the South Limburg outbreak. Seroresponse time of anti‐Coxiella phase II IgM antibodies, that is the period from onset of symptoms to the onset of phase II IgM seroresponse, appears to be extremely variable, ranging from zero to seven months with a median of less than one month (Wielders, Teunis, Hermans, van der Hoek, & Schneeberger, 2015). The same is true for phase II IgG seroresponse. Blood donors from the German cross‐border area were recruited and tested 10–11 months after the peak of abortions on the outbreak farm in South Limburg. Thus, our solitary phase II IgM findings are highly suggestive of recent infections incurred somewhere between mid‐2009 and early 2010, well in line with the South Limburg outbreak, where new cases were reported throughout the entire period from April 2009 to March 2010, with a peak in May 2009. Any link to events predating the South Limburg outbreak can virtually be ruled out in blood donors with solitary phase II IgM response. Blood donors with combined phase II IgM and IgG serology also may be linked to the South Limburg outbreak, although infections incurred earlier cannot be ruled out in these cases. Median half time decay rates of IgM phase II antibodies appear to vary widely, ranging from less than a month to several years. Thus, even solitary phase II IgG findings would fit our hypothesis of a link with the South Limburg outbreak (Teunis et al., 2013).

Seroprevalence in 2010 German blood donors living in the city of Aachen, which borders directly with South Limburg, was more than twice as high as 2008 pre‐outbreak seroprevalence in South Limburg. Since there are no natural or man‐made obstacles standing in the way of airborne transmission between the eastern part of South Limburg and Aachen, any Q fever events on either side of the border would likely impact both areas in similar ways, depending among others on the prevailing wind direction at the time of the outbreak. Conversely, we would expect pre‐outbreak seroprevalence in Aachen not to be higher than in neighbouring South Limburg, suggesting that the higher seroprevalence rate observed in 2010 blood donors may reflect a real increase in Q fever infections.

Overall precision of our data for the German study area was limited. Sample sizes of blood donors per postcode were small, particularly in postcodes located at larger distances from the outbreak farm. For lack of individual blood donor data such as residential address and travel patterns, we had to use postcode centroids as a proxy, resulting in low resolution and possibly misclassification regarding exposure location.

When interpreting seroprevalence and incidence rates in blood donors, one always needs to realize that the study population consists of adult, healthy blood donors, not of randomly selected citizens. However, while donors in many cases poorly represent the general population, infections incurred through airborne transmission are generally independent of donor status, reducing biases caused by the comparison of donors and the general population. A Dutch study among blood donors showed that the age and sex distribution of the study population was very similar to the age and sex distribution of the notified Q fever cases in the Netherlands (Hogema et al., 2012). A recent Australian study found the seroprevalence in blood donors to be lower than in the general population, but indicates that different laboratory methods and population sampling may account for some of the differences (Gidding et al., 2019). Seroprevalence in our group of blood donors also may have underestimated the true seroprevalence in the general population, due to the selection process with excluded donor candidates with signs of acute or recent infection, and those with known risk exposures.

Ideally, our findings should be replicated by serological studies of preserved pre‐ and post‐outbreak human samples from other Dutch–German border regions that likely were affected by the Dutch epidemic in 2007–2010. Meanwhile, in the absence of such studies, our findings argue for intensified and harmonized cross‐border communicable disease control, including public health communication to professionals, public and media, as well as exchange of data suitable for surveillance, detection and early warning. In addition, we urgently recommend that patients, who live in affected areas and have predisposing conditions, serological evidence or clinical symptoms consistent with persistent focalized (chronic) Q fever infection, should be considered for low‐threshold screening, keeping in mind that chronic Q fever may have atypical presentations (Melenotte, Million, & Raoult, 2020).

## ETHICAL APPROVAL

Our study was ethically approved by the medical ethics committee of the Maastricht University Medical Centre (number 104034) and the medical ethics committee of the RWTH Aachen University Hospital (number EK 026‐10) and conforms to internationally recognized standards (Declaration of Helsinki).

## CONFLICT OF INTERESTS

The authors declare they have no conflict of interests.
